# Identification of Differentially Expressed Genes in Spinal Cord Injury

**DOI:** 10.3390/genes16050514

**Published:** 2025-04-28

**Authors:** Andrew Chang, Shevanka Dias Abeyagunawardene, Xiaohang Zheng, Haiming Jin, Qingqing Wang, Jiake Xu

**Affiliations:** 1 Medical School, The University of Western Australia, Perth, WA 6009, Australia; 23014711@student.uwa.edu.au (A.C.); 22964992@student.uwa.edu.au (S.D.A.); 2 Department of Orthopaedics, The Second Affiliated Hospital and Yuying Children’s Hospital of Wenzhou Medical University, Wenzhou 325027, China; 3 Department of Orthopaedic Surgery, Sir Run Run Shaw Hospital, School of Medicine, Zhejiang University, Hangzhou 310016, China; 4 School of Biomedical Sciences, The University of Western Australia, Perth, WA 6009, Australia; 5 Shenzhen Insitute of Advanced Technology, Chinese Academy of Sciences, Shenzhen 518055, China

**Keywords:** spinal cord injury, inflammation, myelin, autoimmune response, immune response, RNA-seq, differentially expressed genes

## Abstract

Background: Spinal cord injury (SCI) remains a profound medical challenge, with limited therapeutic options available. Studies focusing on individual molecular markers have limitations in addressing the complex disease process. Methods: This study utilizes RNA-sequencing (RNA-seq) to investigate the differentially expressed genes (DEGs) in spinal cord tissue from a rat SCI model at 1 and 21 days post-injury (dpi). After data processing and analysis, a series of biological pathway enrichment analyses were performed using online tools DAVID and GSEA. Interactions among the enriched genes were studied using Cytoscape software to visualize protein–protein interaction networks. Results: Our analysis identified 595 DEGs, with 399 genes significantly upregulated and 196 significantly downregulated at both time points. *CD68* was the most upregulated gene at 21 dpi, with a significant fold change at 1 dpi. Conversely, *MPZ* was the most downregulated gene. Key immune response processes, including tumor necrosis factor (TNF) production, phagocytosis, and complement cascades, as well as systemic lupus erythematosus (SLE)-associated pathways, were enriched in the upregulated group. The enriched pathways in the downregulated group were related to the myelin sheath and neuronal synapse. Genes of interest from the most significantly downregulated DEGs were *SCD*, *DHCR24*, *PRX*, *HHIP*, and *ZDHHC22*. Upregulation of Fc-γ receptor genes, including *FCGR2B* and *FCGR2A*, points to potential autoimmune mechanisms. Conclusions: Our findings highlight complex immune and autoimmune responses that contribute to ongoing inflammation and tissue damage post-SCI, underscoring new avenues for therapeutic interventions targeting these molecular processes.

## 1. Introduction

### 1.1. The Burden of Spinal Cord Injury

The spinal cord belongs to the central nervous system (CNS) and houses neuronal circuits that integrate and coordinate complex motor, sensory, and autonomic functions between the brain and body [1]. The spinal cord lies within a meningeal sheath in the vertebral column and is divided into segments longitudinally, with each segment having sensory, motor, and autonomic nerve fibers that leave via a spinal nerve [1]. SCI is the injury of the spinal cord from the foramen magnum to the cauda equina which occurs as a result of incision, compulsion, or contusion [2]. Globally, the most common causes of SCI are traffic accidents, gunshot injuries, knife injuries, falls, and sports injuries [2]. SCI is a devastating neurological state with extensive physical, psychological, and financial complications [3]. The annual global prevalence of SCI is estimated to fall between 250,000 and 500,000 individuals [4], and it is highly prevalent in developed nations, with a high economic burden. In Canada, the calculated annual economic burden of SCI is almost 2.67 billion dollars, with the total lifetime cost estimated to exceed 3 million dollars per person [5]. Currently, the available treatments are highly limited for patients with permanent disability and only provide supportive relief and rehabilitation [4]. SCI treatment limitations are primarily due to a poor understanding of the complex disease pathophysiologic consequences post-injury, especially on a gene-expression level [3].

### 1.2. Pathophysiology of SCI & Regeneration

Our understanding of SCI mechanisms in the past few decades has benefited due to the development of transgenic and preclinical animal models. The pathophysiology of SCI is traditionally categorized into both acute and chronic phases and incorporates a cascade of destructive events, such as ischemia, oxidative stress, inflammatory events, apoptotic pathways, and locomotor dysfunction [3]. More recently, a new systematic classification has been adopted to better define the complicated pathophysiological processes of SCI, which states that the inability of the spinal cord to regenerate can be attributed to a stage-specific imbalance of tissue-, cell-, and molecule-promoting and inhibiting factors in the microenvironment [6]. A transcriptome-wide study can contribute to our understanding of this environment.

#### 1.2.1. Disease Progression

The initial stage of SCI is known as the primary injury, which describes the mechanical injury to the spinal cord. This results in the destruction of neural tissue, disruption of key axonal networks, vascular edema, and disruption of the glial membrane and the blood-spinal cord barrier [3]. Secondary injury describes the cascade of biochemical, mechanical, and physiological changes within neural tissues that ensue [7]. This stage is further sub-classified into acute, sub-acute, and chronic [8]. The timing of these stages remains debated; however, it is generally accepted that the acute phase occurs within the first 48 h, the sub-acute phase extends up to two weeks, and the chronic phase begins thereafter. While these phases overlap, the molecular signals within the injury microenvironment drive variations in cell phenotype. In mice studies, the most dynamic molecular changes occur within 3 days of injury, and by 2 weeks, a second wave of neuroinflammation emerges with neuronal cell changes. Beyond this point, major cell types are significantly deviated from uninjured states, which reflects prolonged alterations in the cellular microenvironment [9,10]. Therefore, a study examining the acute phase and chronic phase of injury allows us to identify key drivers that remain in between the immediate injury and long-term pathological changes, without confounding transitional processes that will not influence chronic outcomes. Chronic outcomes of secondary injury such as axon failure over reactive scar-forming astrocytes and remyelination are crucial to address especially since the main victims of SCI worldwide are well past their moment of injury [11].

#### 1.2.2. Central vs. Peripheral Nervous System

The ability to regenerate relies on neurons transcriptionally reverting to an embryonic-like state [12] and, hence, is associated with specific gene expression or phenotype. Peripheral nervous system (PNS) neurons regenerate more readily than CNS neurons, to the extent that even spinal motor neurons that project to form peripheral nerves regenerate and maintain muscle function post-injury [13]. The CNS and PNS respond differently to injuries, and the differing gene expression post-injury may give insight into the underlying complex biological processes involved. Unlike CNS cells, PNS axons increase their intrinsic growth state due to transcription-dependent changes in their gene expression and axonal transport after a conditioning lesion [14]. It has also been shown that the injury-induced regulation of neuronal transcription in sensory neurons of the PNS relies on the induction of a specific network of genes that promote regeneration, which cannot be seen in the CNS [15]. Since the CNS fails to upregulate certain pro-regenerative genes that are automatically upregulated in the PNS, it logically follows that there could be a combination of downregulated genes in CNS neurons post-injury that are preventing regeneration.

#### 1.2.3. Regenerative Hypotheses

Our understanding of pathological genes contributing to SCI pathology may be incomplete due to a reliance on single-gene hypotheses and protein analyses driving research in the area. Several pioneering studies have hypothesized that both the Nogo and *PTEN* genes are responsible for inhibiting spinal cord regeneration [16,17,18,19]. In addition, there is the glial scar hypothesis, which describes the physical barrier that prevents axonal regeneration [8,20,21,22]. While some studies did show the therapeutic benefit of targeting these domains of injury, these therapies did not restore function in other injury models. Zheng et al. saw no remarkable CST axon regeneration in knockout mice with targeted disruption of the Nogo loci compared to wild-type mice following spinal cord transection [23]. The glial scar theory is also difficult to appreciate as it is initially neuroprotective in SCI recovery. In the acute and sub-acute secondary phase of SCI, the glial scar acts as both a suppressor and regulator of inflammation via immune cells, while also restricting inflammatory and fibrotic infiltrate in the chronic phase [22]. Anderson et al. observed that ablation of the glial scar fails to create spontaneous axonal regrowth [24].

Several studies have also examined single DEGs. The Bcl-2 family of proteins are potent regulators of apoptotic cell death, which include Bax and anti-apoptotic Bcl-2 [25]. Bax expression in vivo correlated with apoptotic neuronal death in complete SCI and was increased in injured spinal cord tissues, with a decrease in Bcl-2 levels [26,27]. Another DEG identified was published in a study by Park et al., who observed a significant fold increase in *SOCS3* mRNA expression in injured spinal cords of mice, with consequent cellular protection after the introduction of a viral vector [28].

### 1.3. Genetic Analysis

**Figure 1:**
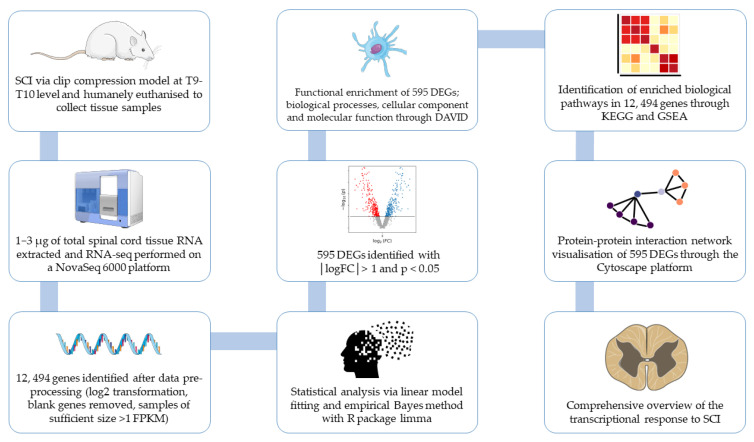
Analytical pipeline and overview of our study design.

These methods, while informative, might not capture the full spectrum of gene expression changes that occur at the RNA level, potentially overlooking critical regulatory mechanisms and novel targets for therapeutic intervention. There is also a sparse amount of research pertaining to the genes that are downregulated in SCI, with no well-established genes. Microarrays were the gold standard for gene expression for over 15 years [29] but have been swapped out in favor of RNA-Seq. RNA-Seq quantifies the levels of various RNA types in a sample by directly sequencing the RNA and counting the number of sequences [30]. Although RNA-Seq is expensive and requires high computational and data-storage capacity, it is useful in exploratory analysis, has a high throughput, and has less background noise and better detection than even microarrays [30]. Unlike microarray studies, which are limited to predefined gene sets, RNA-seq allows for the exploration of the entire transcriptome, capturing a broader spectrum of gene expression changes to enhance our understanding of the intrinsic neuronal and molecular responses to SCI. Although reporting biases of DEGs in the literature exist and gene expression changes may not always translate into consequential biological activity, data from DEG analysis can be pooled with other biological data to create integrated analyses, forming the target landscape of a pathological state [31]. Hence this study design utilizing total cell RNA can be a valuable tool for identifying novel cell types associated with disease, suggesting new pathophysiological mechanisms, uncovering potential drug targets, and discovering potential biomarkers [32]. The figure below illustrates our analytical pipeline that will provide the foundation for future research into gene suppression or supplementation to regenerate the CNS after injury (Figure 1).

## 2. Method

### 2.1. Gene Collection

Female Sprague-Dawley rats (200–220 g) were obtained from the Animal Center of the Chinese Academy of Sciences (Shanghai, China) and housed individually under specific pathogen-free (SPF) conditions in a temperature-controlled facility at the Animal Center of Sir Run Run Shaw Hospital, Zhejiang University. All experimental procedures were approved by the Institutional Animal Care and Use Committee (IACUC) of Zhejiang University (approval number: ZJU20220466) and conducted in accordance with institutional guidelines. Prior to surgery, rats were anesthetized with sodium pentobarbital (40 mg/kg) and the dorsal area was shaved. A clip-compression model of spinal cord injury (SCI) was established at the T9–T10 vertebral level using 5-mm surgical forceps (FT190T, B. Braun, Melsungen, Germany) applied for 1 min. To ensure consistent injury severity, the forceps tips were fully inserted on either side of the exposed spinal cord. Postoperative care included manual bladder expression and shell placement. At 1 and 21 days post-surgery, three rats were humanely euthanized for collection of total spinal cord tissue samples. 

### 2.2. RNA-Sequencing

Total RNA from spinal cord tissue was extracted according to the manufacturer’s instructions using TRIzol reagent. Sample purity was evaluated using a NanoPhotometer (IMPLEN, Westlake Village, CA, USA), while RNA concentration and integrity were assessed with an Agilent 2100 RNA Nano 6000 assay kit (Agilent Technologies, Santa Clara, CA, USA). For library preparation, 1–3 µg of RNA per sample was used. Sequencing libraries were constructed using the VAHTS Universal V6 RNA-seq Library Prep Kit for Illumina (NR604-01/02) according to the supplier’s protocol, with unique index codes assigned to each sample. Preliminary quantification of the RNA libraries was performed using the Qubit RNA Assay Kit on a Qubit 3.0 fluorometer, and samples were diluted to a final concentration of 1 ng/μL. Library insert size was determined with the Agilent Bioanalyzer 2100 system. Once the insert size met the required specifications, the library’s effective concentration (>10 nM) was precisely quantified using a Bio-Rad CFX96 real-time PCR system. The clustering of the index-coded samples was performed on a cBot cluster generation system using HiSeq PE Cluster Kit v4-cBot-HS (Illumina, San Diego, CA, USA) according to the manufacturer’s instructions. Finally, cluster generation and sequencing were performed on a NovaSeq 6000 S4 platform, using the NovaSeq 6000 S4 Reagent kit V1.5 (Illumina, San Diego, CA, USA). The resulting dataset included almost twenty thousand identified genes.

### 2.3. Data Pre-Processing

The expression profile data were processed by log2 transformation. All blank genes were removed from the dataset. The pre-injury control (Con), day 1 post-injury (1 dpi), and day 21 post-injury (21 dpi) samples were filtered to include those of sufficient size (≥1 FPKM). This arbitrary cutoff was applied to remove noise from weakly expressed genes.

### 2.4. Statistical Analysis

The expression profile data were then subjected to multiple linear regression using the lmFit function, immediately followed by further computation with the eBayes function. Finally, the R 4.4.1 package Limma (Linear models for microarray data) was utilized to perform differential gene analysis on the expression profiles. Log fold-change values (logFC) were calculated at 21 dpi and 1 dpi, in comparison to Con. DEGs were defined at 1 dpi and 21 dpi as expressing a log FC magnitude of >1 and were statistically significant (*p* < 0.05). A volcano plot was created of all the statistically significant DEGs, both up- and downregulated, using the ggplot2 package of RStudio 2024.04.2 + 764 [33]. A new dataset was extracted from our filtering to create a list of sustained upregulated and downregulated DEGs; genes that expressed a significant fold change both at 1 dpi and 21 dpi. RStudio was used to generate a heatmap of the top 30 sustained upregulated and downregulated DEGs, an arbitrary cutoff for this visualization. A paired *t*-test was conducted to assess for significant differences between DEGs at 1 dpi and 21 dpi and comparative boxplots were plotted for the DEGs. Individual genes were selected to confirm pathological genes and add to the literature. Box plots were generated within ggplot2 v3.5.1 in R studio [33]. Genes were selected based on the subsequent pathway analysis and their potential for therapeutic benefit.

### 2.5. Functional Enrichment Analysis of Downregulated DEGs

A Gene Ontology (GO) analysis of the upregulated DEGs was conducted using the Database for Annotation, Visualisation, and Integrated Discovery (DAVID) (https://david.ncifcrf.gov/) [34,35]. The analysis identified the top enriched biological processes (GO: BP), cellular components (GO: CC), and molecular functions (GO: MF), with results imported into RStudio for the production of bubble plots which exhibit the enrichment score as well as pathway-related genes associated with the top GO pathways (including BP, CC and MF). A Kyoto Encyclopedia of Genes and Genomes (KEGG) pathway analysis of the DEGs was conducted using the Java-based Gene Set Enrichment Analysis (GSEA) [36]. GSEA was performed in preranked mode using GSEA software version 4.3.3. Genes were ranked according to log fold change. Gene identifiers from *Rattus norvegicus* were mapped using the Mouse_Gene_Symbol_Remapping_Human_Orthologs_MSigDB.v2024.1.Hs.chip file to align with human orthologs. Enrichment analysis was conducted against the KEGG pathway gene sets (C2:CP:KEGG_legacy) from the Molecular Signatures Database (MSigDB v2023.2, human, symbols.gmt). Enrichment plots were produced with normalized enrichment scores (NES) and false discovery rates (FDR). These results were transferred to RStudio and represented as bar plots which exhibit the enrichment score as well as the Q-value associated with the core KEGG pathways.

### 2.6. Protein–Protein Interaction (PPI) Network and Module Analysis

The Search Tool for the Retrieval of Interacting Genes (STRING) database was used to generate a PPI network for the DEGs with an interaction score exceeding 0.4 [37]. The STRING output was processed through the Molecular Complex Detection (MCODE) module in Cytoscape software v3.10.2 [38]. Cluster analyses of the PPI network for DEGs were produced. The cluster analyses were conducted using parameters of node score cut-off = 0.2, degree cut-off = 2, k-score = 2, and max. Depth = 100. The interactions of critical proteins are indicated by the number of interactions and the thickness of the lines. The clustered genes underwent further KEGG pathway analyses using DAVID with FDR-corrected *p* < 0.05 used as the level of significance.

## 3. Results from Total Spinal Cord Tissue of Ratticus Norvegicus

The statistical analysis employed in this study was designed to systematically identify DEGs and elucidate the molecular mechanisms underlying SCI. The DEGs, once identified, were used to explore their own functional significance through GO and KEGG. Finally, a cluster analysis through the Cytoscape MCODE module enabled the identification of key regulatory networks. Overall, this formed a sound methodological framework to facilitate the identification of key genes and pathways involved in SCI, addressing the study objective of understanding the molecular landscape of injury and recovery. By integrating differential expression analysis, pathway enrichment, and network analysis, this study provides a comprehensive view of the transcriptional response to SCI, laying the foundation for future research into potential therapeutic interventions.

### 3.1. Differential Gene Expression Analysis Between SCI and Control Groups

**Figure 2:**
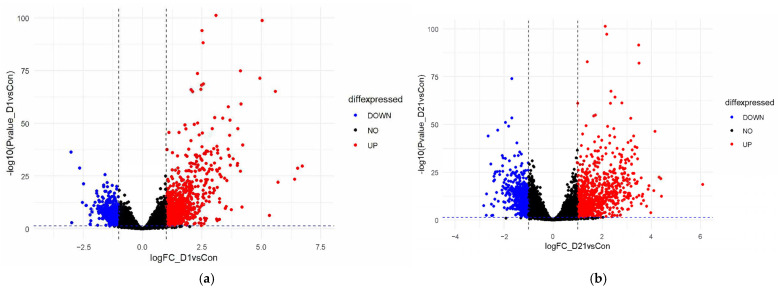
Volcano plot representing temporal gene expression at (**a**) 1 dpi (**b**) 21 dpi.

**Figure 3:**
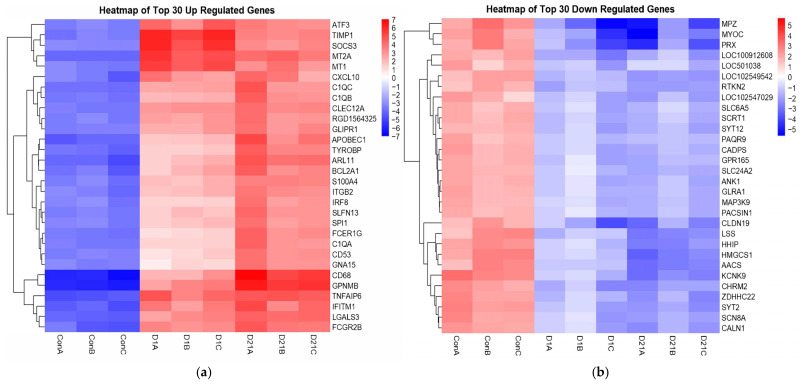
Heatmap based on the hierarchical clustering of the top 30 DEGs ranked according to the 21 dpi log FC in the (**a**) upregulated group and (**b**) downregulated group.

**Figure 4:**
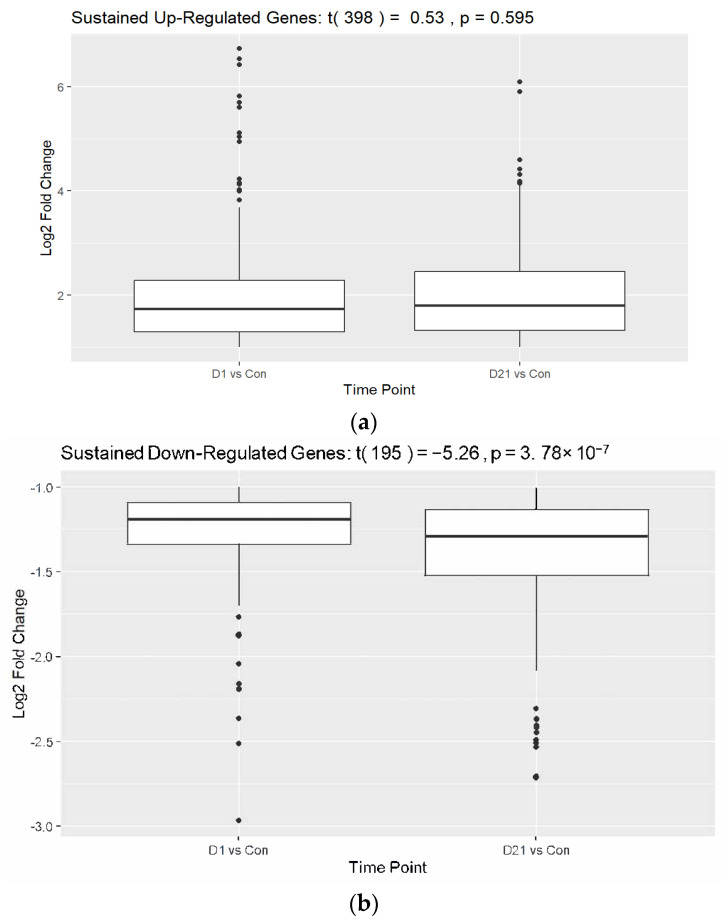
Paired *t*-test analysis of DEGs between 21 dpi and 1 dpi for the (**a**) upregulated DEGs and (**b**) downregulated DEGs.

After filtering and quality control were applied, our data were reduced to 12,494 genes (all samples >1 FPKM, zero values removed). Our analysis identified 864 significantly upregulated and 586 significantly downregulated genes at 1 dpi. We identified 873 upregulated and 553 downregulated genes at 21 dpi (Figure 2). When considering both time points simultaneously, we identified 595 DEGs between the injury and control groups, with 11,899 genes being insignificant based on the following parameters: Absolute Value (21 dpi/Con logFC AND 1 dpi/Con logFC) > 1 and *p* < 0.05. Of the 595 genes, 399 were upregulated and 196 were downregulated at 1 dpi and 21 dpi (Table S1). The top 30 DEGs of each group were presented in a heatmap with hierarchical clustering (Figure 3), and a paired *t*-test analysis between timepoints was performed. The downregulated group revealed a significant difference between the mean in 21 dpi versus 1 dpi, with the former group being significantly lower, t(195) = −5.26, *p* < 0.001 (Figure 4). This result suggests that genes affected by injury fail to adapt to the injured state. The *t*-test showed no significant change in the upregulated group, which suggests that total RNA expression had no significant variation at each time point for upregulated DEGs.

### 3.2. Analysis of Established Genes Involved in SCI

**Figure 5:**
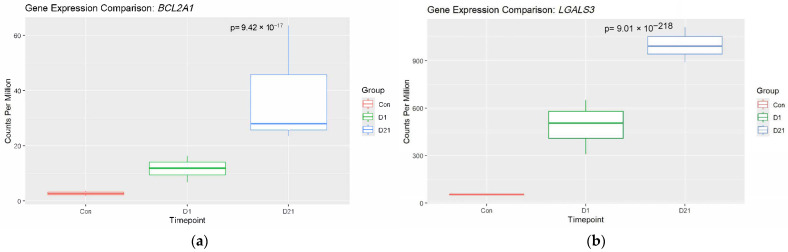

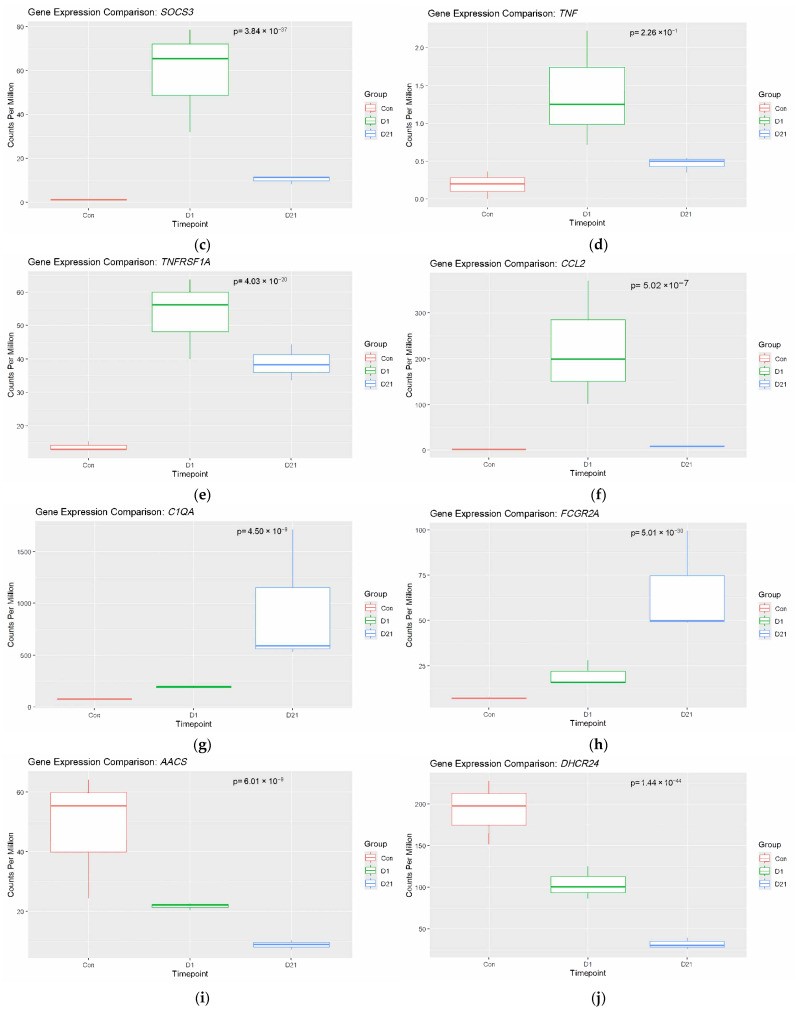

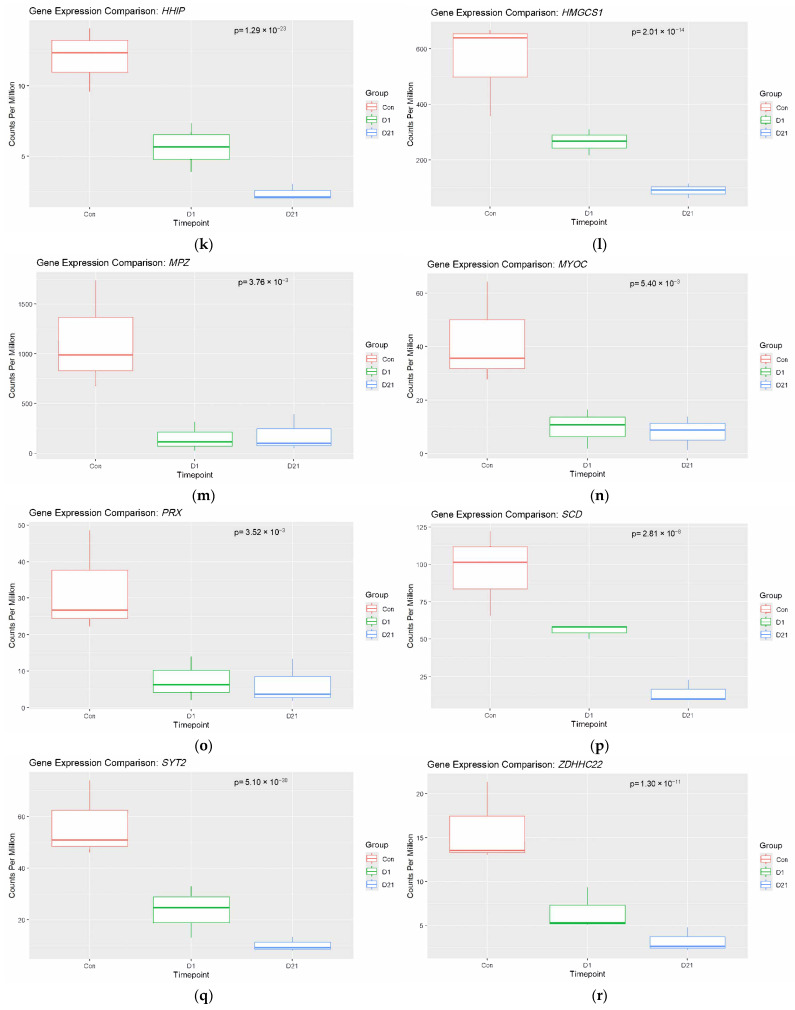
Temporal RNA expression changes confirming established genes in the literature, as well as novel targets in the downregulated group: (**a**) *BCL2A1*, (**b**) *LGALS3*, (**c**) *SOCS3*, (**d**) *TNF*, (**e**) *TNFRSF1A*, (**f**) *CCL2*, (**g**) *C1QA*, (**h**) *FCGR2A*, (**i**) *AACS*, (**j**) *DHCR24*, (**k**) *HHIP*, (**l**) *HMGCS1*, (**m**) *MPZ*, (**n**) *MYOC*, (**o**) *PRX*, (**p**) *SCD*, (**q**) *SYT2*, and (**r**) *ZDHHC22*.

DEG results for several genes known to have a role in SCI pathogenesis were checked in our data to provide confidence in the injury model. These included Suppressor of Cytokine Signaling 3 (*SOCS3*), B-Cell Lymphoma 2 (*BCL2*), *LGALS3*, and *TNF*. All these genes were found to be significantly upregulated in the injury group at 1 dpi with logFC ≥ 1 and *p* < 0.05 (Figure 5a–d). Additional box plots were generated for genes of interest that were likely to offer therapeutic benefit for both groups (Figure 5e–r).

### 3.3. Enrichment Analysis of DEGs

**Figure 6:**
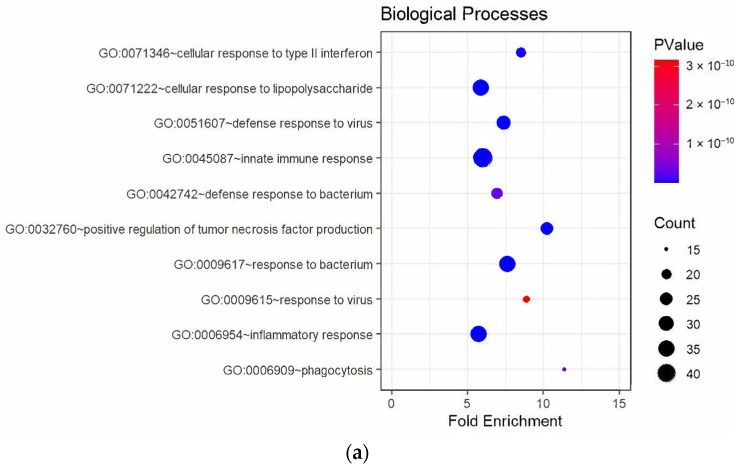

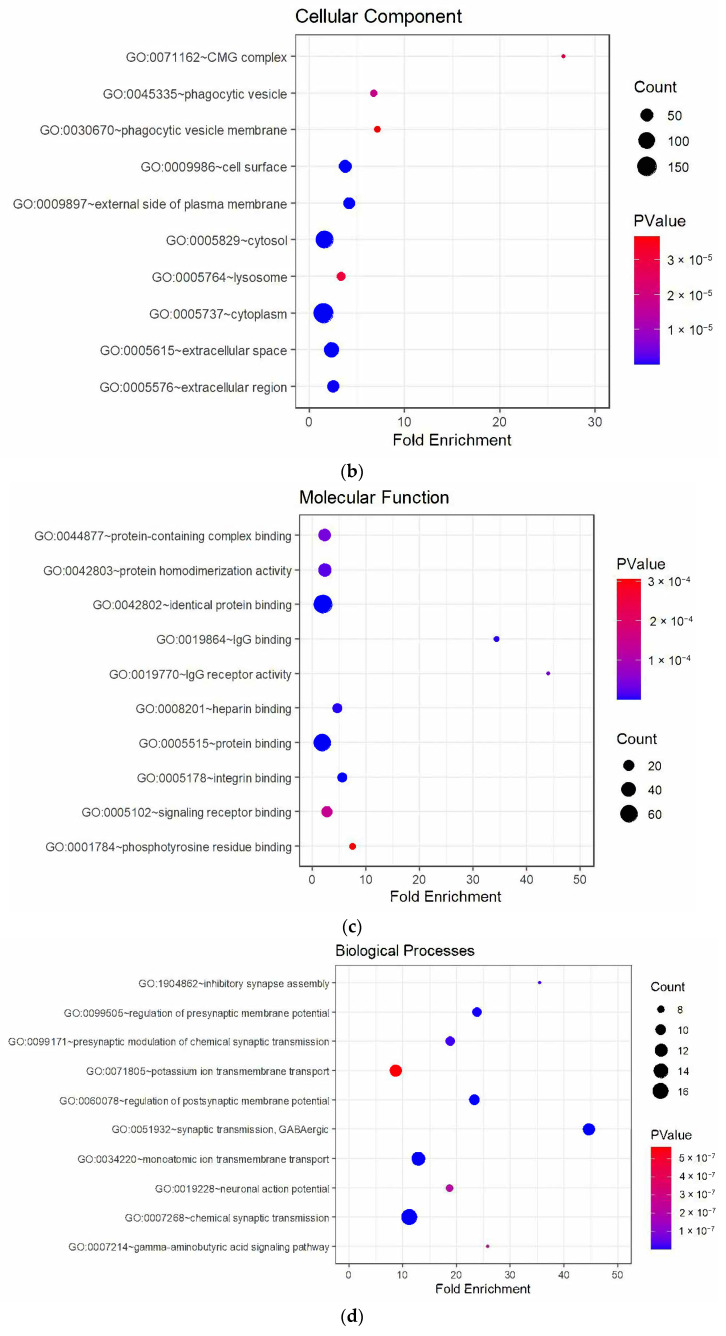

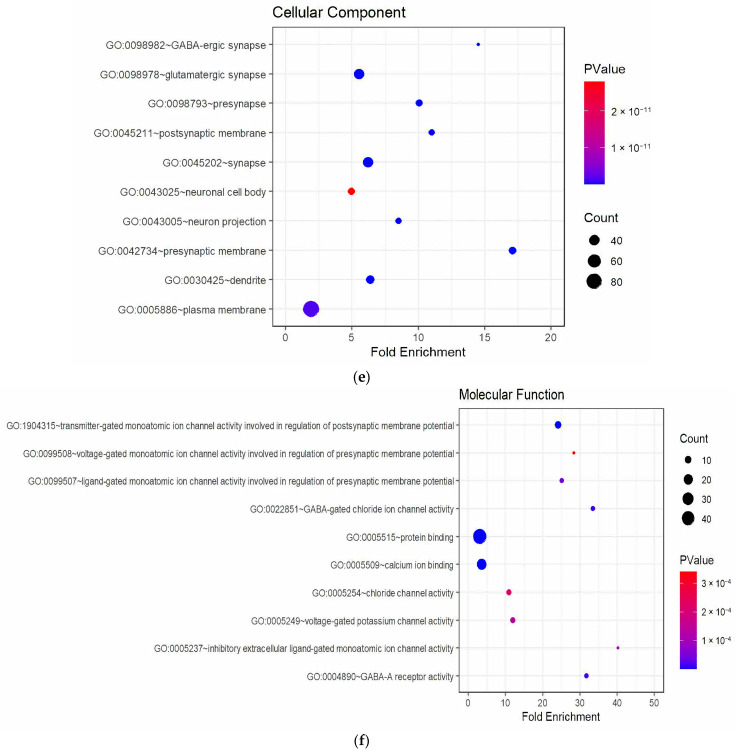
Gene ontology analysis with the top 10 enrichment terms plotted on bubble plots for the upregulated group (**a**–**c**) and the downregulated group (**d**–**f**).

Using DAVID software (version DAVID 2021), the top enriched biological processes identified for the upregulated genes are the positive regulation of tumor necrosis factor production, phagocytosis, inflammatory response, innate immune response, cellular response to lipopolysaccharide, and cellular response to type II interferon (Figure 6a and Table S2). The top enriched cellular components are the CMG complex, phagocytic vesicle, phagocytic vesicle membrane, cytoplasm, and cytosol (Figure 6b and Table S2). The top enriched molecular functions are IgG binding, IgG receptor activity, protein binding, signaling receptor binding, and phosphotyrosine residue binding (Figure 6c and Table S2).

Downregulated genes were also used to identify processes that were affected by SCI. The top enriched biological processes (that is, GO terms that were most affected after injury) included those related to the synapse: synaptic transmission, inhibitory synapse assembly, monoatomic ion transmembrane transport, potassium ion transmembrane transport, and neuronal action potential (Figure 6d and Table S3). The top enriched cellular components also involved those related to the synapse, including the presynaptic membrane, GABAergic synapse, plasma membrane, and glutamatergic synapse (Figure 6e and Table S3). The top enriched molecular functions revolved around channel activity, including inhibitory extracellular ligand-gated monoatomic ion channel activity, GABA-gated chlorine ion channel activity, calcium ion binding, and protein binding (Figure 6f and Table S3).

**Figure 7:**
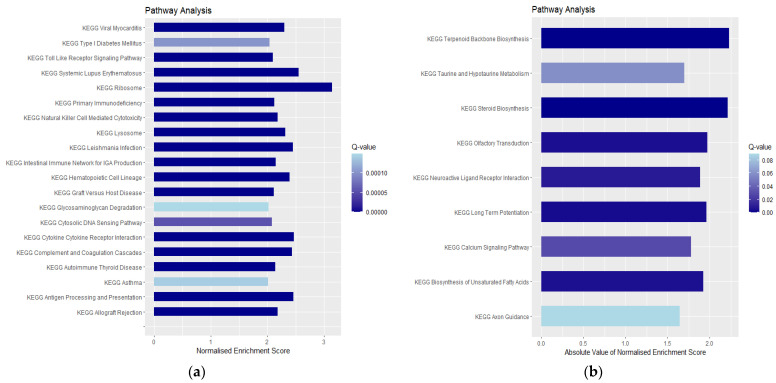
The top KEGG pathways enriched in (**a**) upregulated gene sets and (**b**) downregulated gene sets.

**Figure 8:**
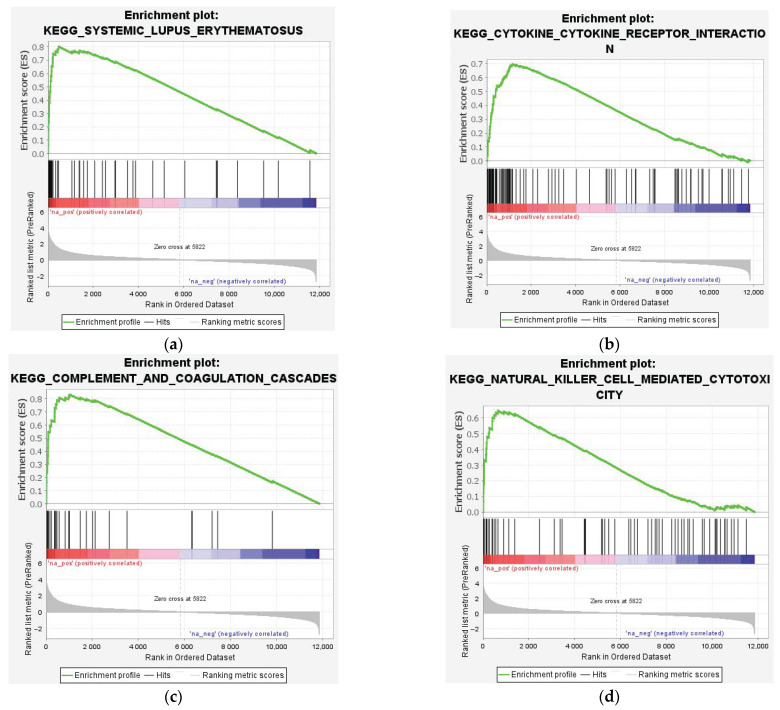

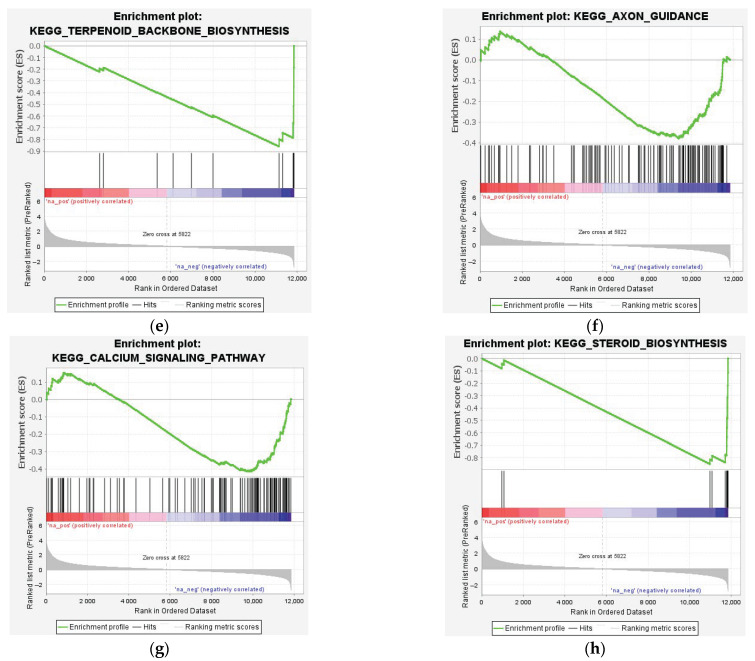
Enrichment plots for key KEGG pathways highlighted in the upregulated group, including (**a**) systemic lupus erythematosus (**b**) cytokine–cytokine receptor interaction (**c**) complement and coagulation cascades (**d**) natural killer cell-mediated cytotoxicity. Pathways enriched in the downregulated group include (**e**) terpenoid backbone synthesis (**f**) axon guidance (**g**) calcium signaling pathway (**h**) steroid biosynthesis.

GSEA pathway analysis identified 186 gene sets. 113 gene sets were upregulated and 73 gene sets were downregulated. Upregulated genes were enriched in the ribosome, cytokine–cytokine receptor interaction, lysosome, systemic lupus erythematosus, and complement and coagulation cascades (Figure 7a and Figure 8a–d; Table S4). Specific genes involved in autoimmunity related to these processes, including immunoglobin-involved FCGR2A and complement C1QA, were plotted (Figure 5g,h). Our results point to an enrichment of autoimmune processes after SCI, likely due to the disruption of the blood-spinal cord barrier. The downregulated genes were enriched in calcium signaling, neuroactive ligand–receptor interaction, and the biosynthesis of terpenoid backbones, steroids, and unsaturated fatty acids (Figure 7b and Figure 8e–h; Table S4).

### 3.4. PPI Interaction Network Analysis

**Figure 9:**
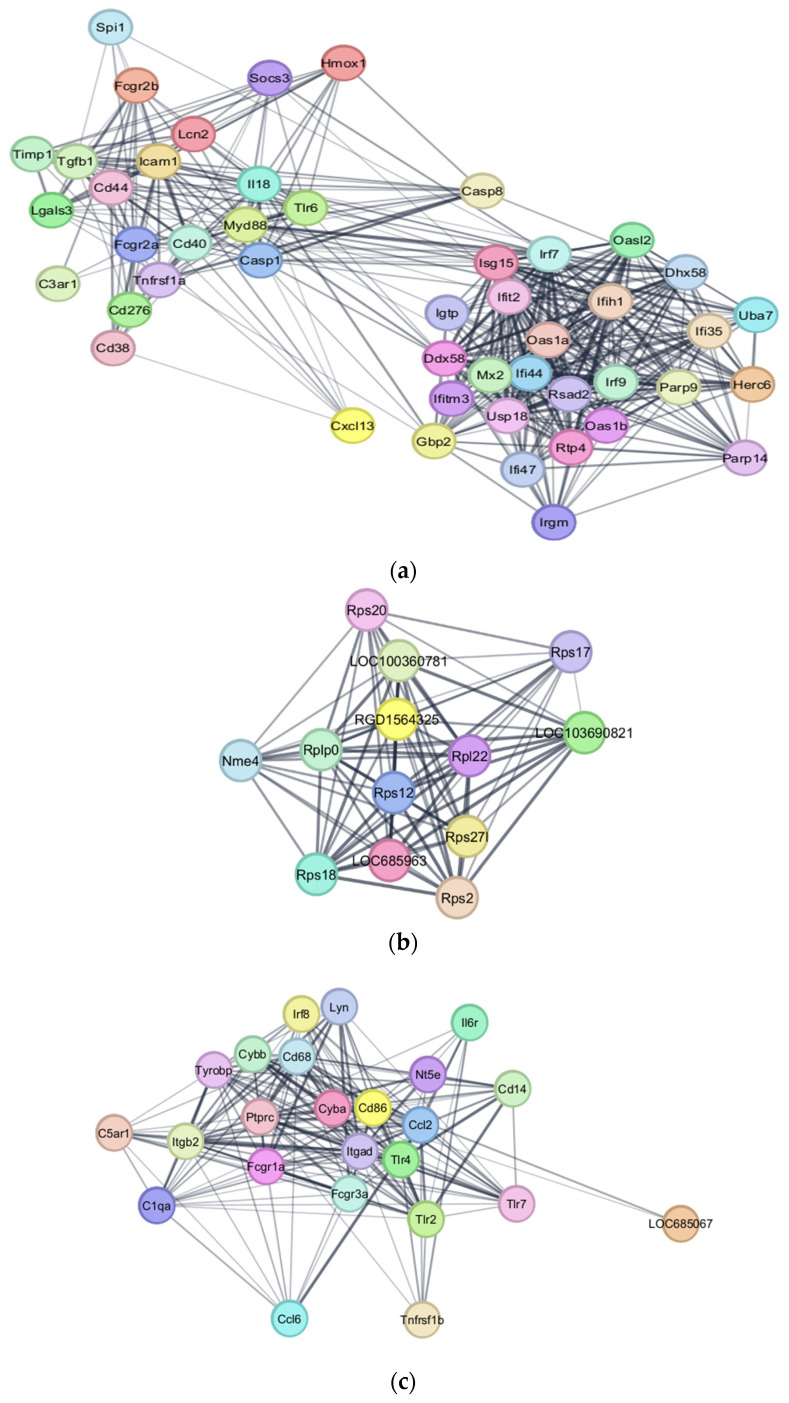

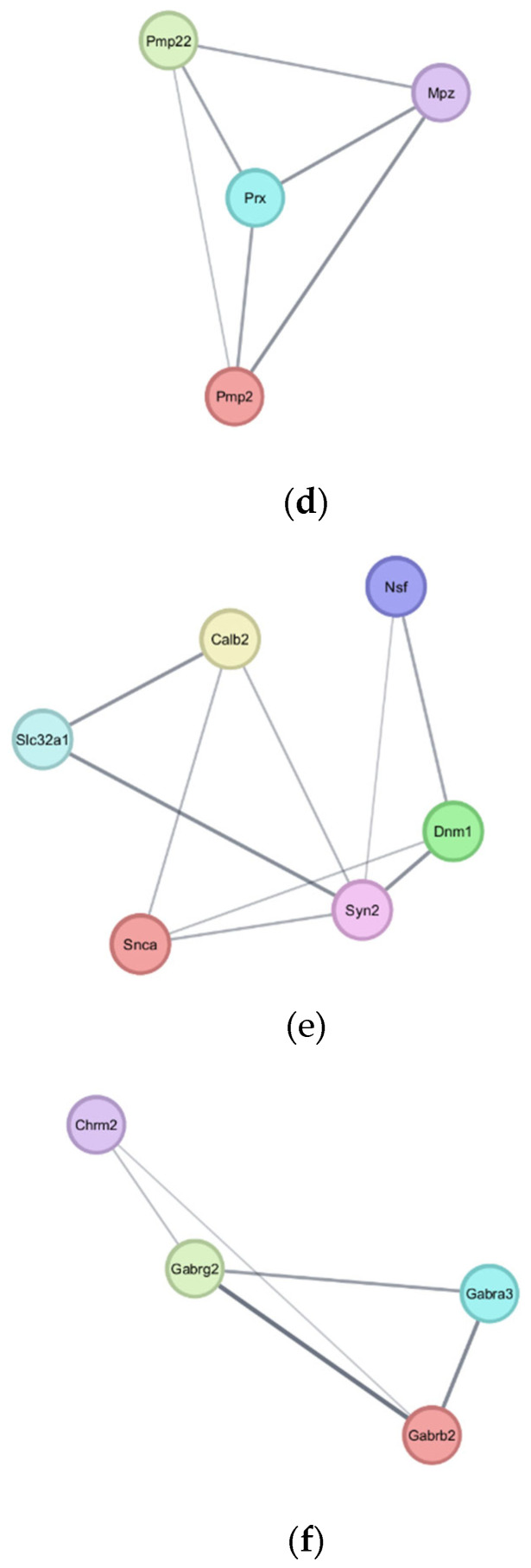
Visual representation of associated protein networks of identified gene clusters. Upregulated DEGs are visualized in (**a**) Cluster 1, (**b**) Cluster 2, and (**c**) Cluster 3. Downregulated DEGs are visualized in (**d**) Cluster 1, (**e**) Cluster 2, and (**f**) Cluster 3.

PPI network analysis identified the most inter-connected genes in our DEGs. The top gene clusters in the upregulated group were significant with MCODE scores of 18.696, 15.043, and 13.000. The gene file was input into DAVID to generate their associated KEGG pathways. Cluster 1 genes were associated with the TNF signaling pathway, NF-kappa B signaling pathway, RIG-1-like receptor signaling pathway, and Nod-like receptor signaling pathway (Figure 9a and Table S5a). Cluster 2 genes were associated with neutrophil extracellular trap formation, phagosome, lipid and atherosclerosis, and complement and coagulation cascades (Figure 9b and Table S5a). Cluster 3 genes were associated with ribosome pathways (Figure 9c and Table S5a).

The top gene clusters in the downregulated group were also identified, with MCODE scores of 4.0, 3.6, and 3.33, respectively. However, the first cluster was not statistically significant (Figure 9d). Cluster 2 genes were associated with synaptic vesicle and GABAergic synapse pathways (Figure 9e and Table S5b). Cluster 3 genes were associated with nicotine and morphine addiction, neuroactive ligand–receptor interaction, GABAergic synapse, and retrograde endocannabinoid signaling (Figure 9f and Table S5b).

## 4. Discussion

In this study, we studied the molecular and cellular pathways involved in SCI through differential gene expression analysis as determined by the temporal expression of total spinal cord mRNA. A total of 12,494 genes were identified in the spinal cords of Ratticus Norvegicus species after data pre-processing. After filtering and quality control were applied, we identified 399 and 196 genes that exhibited significantly sustained upregulation and downregulation at 1 and 21 dpi, respectively. There has been substantial research focused on the identification of upregulated genes following SCI to elucidate potential pathological mechanisms. However, comparatively less attention has been directed toward investigating the downregulated genes in this context. Genes that were significantly downregulated at 1 dpi reveal those that were immediately affected by SCI. However, the host may naturally correct these deficits without the need for any intervention. The genes that were also significantly downregulated at 21 dpi suggest that they are non-temporarily affected by SCI and the host is unable to mount a recovery at the molecular level. Since SCI results in non-temporary neurological deficits, it logically follows that the sustained downregulated DEGs may prove to be useful targets with respect to reversing the negative effects of spinal cord damage. The paired *t*-test analysis of the sustained downregulated DEGs at 21 dpi versus 1 dpi (Figure 4b) supports this hypothesis as it revealed a statistically significant further downregulation of the genes [t(195) = −5.26, *p* < 0.001]. This suggests that the significantly downregulated genes will continually decline and fail to mount a regenerative response. Comparatively, the same test performed on the upregulated DEGs shows little deviation of the mean RNA expression between the two groups at 21 dpi and 1 dpi, suggesting that post-injury, the pathological state that ensues remains constant. However, this result is not statistically significant.

### 4.1. Upregulated Genes and Neuroinflammation

#### 4.1.1. Microglia Activity After SCI

It is well known that following insult to the spinal cord, there is a cascade of neuroinflammation due to the innate immune response that is mediated by cytokines and chemokines released by resident microglia, astrocytes, and peripherally derived immune cells [39]. The DEG with the highest mRNA expression at 21 dpi was *CD68* (Table S1). *CD68* is a transmembrane glycoprotein highly expressed by cells of the monocyte lineage and tissue macrophages [40]. These cells display both structural and functional heterogeneity that are adaptive within their local microenvironment and can play roles in both physiological and pathological processes, including inflammation and auto-immunity [40,41]. Microglia are a subset of tissue macrophages that populate the central nervous system and are the main effectors of the inflammatory response after SCI in addition to macrophages derived from the peripheral circulation due to homeostatic insult [42]. Macrophages have historically been described to exist in two states, resting and activated [43]. Activated macrophages are broadly classified into two main groups; classically activated M1 and alternatively activated M2 [44]. In the CNS M1 macrophages are thought to be neural destructive, and M2 macrophages are thought to be neural protective [43,45]. However, macrophage reactivity is not binary, and intermediate morphologies that exist on a continuum complicate our understanding of their dual role in both inflammation and recovery [46]. Furthermore, peripheral macrophages are difficult to distinguish from microglia by immunohistology and antigenic markers alone [42]. Nonetheless, it has been shown that macrophage/microglia anti-inflammatory cytokines are prominent at 1-week post-injury and pro-inflammatory effects persist after 1 week for up to months [47].

*CCL2* exhibited the highest mRNA expression in the acute phase of injury 1 dpi in our model (Figure 5f). *CCL2* is released by resident astrocytes to activate microglia, which contributes to the perpetual formation of the glial scar, which consists of peripheral monocytes, reactive microglia, and a dense astrocytic scar border. The role of the glial scar is controversial. Destruction of the glial scar has been shown to worsen locomotor outcomes and aggravate neuroinflammation [48]. However, it is also a physical barrier to axon regeneration. One study showed that the reduction of *CCL2*-recruited microglia at the site of injury led to remarkably improved locomotor function in rats [49]. It is likely that the interaction of *CCL2* and *CCR2* could activate several signaling cascades, including PI3K/Akt/ERK/NF-κB, PI3K/MAPKs, and JAK/STAT-1/STAT-3, contributing to SCI pathogenesis [50].

Lipocalin 2, also known as neutrophil gelatinase-associated lipocalin (*LCN2*), also exhibited a significant mRNA expression at 1 dpi. *LCN2* has diverse functions and is important for both cellular apoptosis and survival. It is widely expressed in microglia and its expression has been shown to be strongly enhanced by inflammatory stimulation [51]. Its role in microglia is largely considered to be proapoptotic, acting in an autocrine manner to induce amoeboid transformation of microglia rendering vulnerability to other apoptotic signals. Tong et al. demonstrated that induction of *LCN2* does indeed correlate with apoptosis; however, they concluded that it is also a survival factor against toxic stimuli [52]. One study demonstrated suppression of *LCN2* inhibited the activation of neurotoxic microglia and astrocytes [53].

#### 4.1.2. Microglia and the Complement System

Emerging research has identified CD68 and complement C1q as potential markers for the identification of reactive microglia in the CNS [54]. C1q expression in resting microglia is often low, however, increases dramatically in reactive microglia [55]. We confirmed this as expressions of *C1QA*, *C1QB*, and *C1QC* were among the highest DEGs in our analysis 21 dpi and the complement pathway has been identified in both KEGG analysis and protein association networks (Figure 5g, Figure 7a and Figure 8c). *C1QA*, *C1QB*, and *C1QC* encode for the respective chains of the C1q protein, which is organized into six heterotrimeric subunits. Expression of C1q has also been found to remain high after blood-brain-barrier (BBB) dysfunction is restored in a BBB breakdown rat model [55]. Complement and coagulation cascades were an upregulated KEGG pathway as well as played a significant role in our protein network analysis, suggesting that both prolonged microglia/macrophage reactivity and its synergism with complement activation are imperative to the mechanisms of sustained injury in SCI pathology.

#### 4.1.3. Tumor Necrosis Factor and Associated Pathways

The role of tumor necrosis factor α (TNF-α) has been widely studied. Within hours of SCI, local immune cells release inflammatory cytokines IL-1, IL-6, and TNF that are all upregulated and continue to be over 14 dpi [56]. Among the top enriched biological processes in our study were positive regulation of tumor necrosis factor production, innate immune response, and inflammatory response. TNF was not identified as a DEG and was removed by the filtering process as its mRNA expression levels did not meet average FPKM cutoff requirements, although log fold changes were significant >1 (Figure 5d). *TNFAIP6*, *TNFRSF1B*, and *TNFRSF1A* are genes that are regulated by TNF-α that have been identified as DEGs. TSG-6 is the protein product of *TNFAIP6* that is upregulated after inflammation and participates in the negative regulation of inflammatory response, generally exhibiting tissue-protective properties, but has at times been correlated with disease pathology [57]. Within the CNS, TSG-6 is up-regulated after injury and binds to hyaluronan (HA) to form an HA-rich glial scar that coalesces at the site of primary injury, contributing to an immunosuppressive environment [58,59]. The change in expression of *TNFRSF1A* was higher than *TNFRSF1B* expression at one day post-injury however was slightly lower at twenty-one days post-injury (Figure 5e). *TNFRSF1A* encodes for the TNFR1, a death domain-containing receptor, and its activation leads to both apoptosis and necroptosis, whereas TNFR2, encoded by *TNFRSF1B*, promotes cell survival [60]. TNFR1 is expressed ubiquitously in all cells, while TNFR2 is found in neurons, oligodendrocytes, regulatory T cells, and monocytes [61]. TNFR2 can protect neurons and oligodendrocytes against neurotoxic insults in vitro, while TNFR1 promotes neurotoxicity and exacerbates axonal damage through pro-inflammatory effects in vitro [62].

### 4.2. Autoimmunity and Immune Dysregulation

Several pathways the analysis has identified include those associated with immune dysregulation and autoimmune disorders, including systemic lupus erythematosus (Figure 7a and Figure 8a). The top enriched molecular functions included IgG binding and IgG receptor activity (Figure 6c). Previous studies have highlighted the complex co-existence of both immune dysregulation and autoimmunity after SCI [63]. Thus, SCI can be characterized by a low-grade inflammatory state due to the bidirectional communication between the nervous, endocrine, and immune systems [64]. Furthermore, the immune system is under neuro-modulatory control via the innervation of lymphoid tissues by the sympathetic nervous system, and SCI would expect to induce level-dependent immune suppression due to the disruption of sympathetic neurons [65]. Traumatic SCI damages the blood–brain–spinal cord barrier and exposes the spine parenchyma to the cells of the adaptive immune system. Foreign CNS antigens can then trigger autoimmune responses, and the production of autoantibodies has been shown to worsen pathology within the spinal cord, with pathological mechanisms comparable to multiple sclerosis [66].

#### Fc-γ Receptors and Autoantibody Production

Ankeny and his colleagues first demonstrated that SCI induces chronic systemic and intraspinal B cell activation with a systemic, lupus-like autoimmune response [67]. Systemic lupus erythematosus (SLE) is characterized by an aberrant immune response leading to autoantibody production and autoreactive lymphocytes that contribute to the activation of innate immune cells via Fc-γ receptor along with complement fixation [68]. Fc-γ receptors are encoded in humans by the FCGR family of genes, and bind to the Fc portion of IgG, serving as a crucial link between humoral and cell-mediated immune responses [69]. Interestingly, genes *FCGR2B*, *FCGR2A*, *FCGR3A*, and *FCGR1A* were among the upregulated genes identified in the analysis (Figure 5h; Table S1). The Fc-γ receptor IIb is the only inhibitory Fc-γ receptor [70]. Our analysis showed a greater than 3- and 4-fold change in *FCGR2B* and *FCGR2A* expression at 1 and 21 dpi, respectively. Our findings disagree with Yan and colleagues, who found a decrease in *FCGR2B* expression at 7 and 14 dpi [71]. However, research on *FCGR2B* in the CNS is rare, and warrants further investigation. Likewise, the role of other novel factors that regulate CNS such as TAFA chemokine like family member 4 (TAFA4) and its cell surface receptor formyl peptide receptor 1 (FPR1) remains to be explored during SCI recovery [72,73].

### 4.3. Downregulated Pathways and Regenerative Hypotheses

A meta-analysis of human and mouse astrocytes in a neurodegenerative CNS disorder identified the downregulated pathways of synaptic integrity, glutamate uptake, and other neuronal support processes [74]. Although this does not relate directly to traumatic spinal cord injuries, it forms the extent of available CNS degeneration sequencing data. Synapse formation and channel activity are key postulated components of CNS axonal regeneration that are required from a neuro-plasticity standpoint [75,76]. Glial cells such as peri-synaptic Schwann cells at the neuromuscular junction have been shown to guide axon regeneration, highlighting the importance of synaptic activity on changes in synapse states and the integrated nature of neurotransmission and long-term synaptic stability [77]. Although these cells may play a role in maintaining synaptic efficiency and promoting synaptic connections after denervation in the PNS, their role in the CNS post-injury is ineffective, but may prove to be a therapeutic target [77]. Synaptic pathways were also identified in the protein–protein interaction analysis as the predominant pathways composing both Cluster 2 and Cluster 3, namely synaptic vesicle cycle, GABAergic synapse pathways, neuroactive ligand–receptor interaction, and retrograde endocannabinoid signaling pathways (Figure 9d–f). It was also found to be a minor component of the top nine downregulated GSEA functional enrichment gene sets, namely, neuroactive ligand–receptor interaction, long-term potentiation, and calcium signaling pathway (Figure 7b).

Our GSEA functional enrichment analysis also found downregulated gene sets that are strongly related to the components required to maintain a healthy myelin sheath; biosynthesis of terpenoid backbones, steroids, and unsaturated fatty acids (Figure 7b). The nervous microenvironment has been postulated to be an essential component in regenerating CNS neurons. Myelin is produced by oligodendrocytes in the CNS and Schwann cells in the PNS. It is an essential component of nervous tissue, maintaining a healthy intracellular electrical and metabolic microenvironment [78]. When combined with a healthy human Schwann cell graft, an injured rat spinal cord was able to demonstrate regeneration, even beyond the graft [79]. According to the literature, the transplantation of stem cells appears to be the most effective strategy for remyelination, by constructing a microcellular environment that promotes myelin sheath generation [3,80,81].

### 4.4. Key Downregulated Genes and Implications

As mentioned previously, downregulated genes are less frequently studied, as they are challenging to interpret and target therapeutically. The top five downregulated genes based on logFC values at 21 dpi were *MPZ*, *HMGCS1*, *SYT2*, *AACS*, and *MYOC*. Other genes of note amongst the top sustained downregulated DEGs were *SCD*, *DHCR24*, *PRX*, *HHIP*, and *ZDHHC22* (Figure 5i–r). In particular, *MYOC* (Myocilin), has an unknown clinical significance with respect to the general CNS [82]. Interestingly, astrocytes forming the outer border of the CNS, the glia limitans superficialis, have an atypical morphology and are identifiable by a single genetic marker, *MYOC*, and may prove to be a therapeutic target to reduce peripheral inflammatory effects on the CNS [83].

#### 4.4.1. Myelination and Lipid Metabolism

*MPZ* (Myelin Protein Zero) is a gene regulating the early developmental stages of Schwann cells and plays a role in their remyelination in the PNS [84]. Although it has been established as a factor required for PNS regeneration, *MPZ* has not been confirmed as a gene required for CNS regeneration. It has, however, been found to be expressed when Schwann Cells were cocultured with induced pluripotent stem cell-derived motor neurons [85]. One study identified the myelination genes of *MPZ*, *PRX*, *PMP2*, and *PMP22*, as downregulated in mouse lumbar spines after a de-loading event [86], all of which were identified as significantly downregulated at both 1 and 21 dpi in this study. *HMGCS1* (3-Hydroxy-3-Methylglutaryl-CoA Synthase 1) is a potential regulator of the mevalonate pathway, affecting cholesterol synthesis and myelin sheath formation [87,88]. One study found that *HMGCS1* was downregulated along with other cholesterol-related genes in rats post-SCI; however, it was not identified as a key individual gene [89]. *SCD* (Stearoyl-CoA Desaturase) is responsible for the biosynthesis of monosaturated fatty acids from saturated fatty acids, which may, in turn, play a neurotrophic role [90]. *DHCR24* (24-Dehydrocholesterol Reductase) is a gene responsible for the final step of cholesterol biosynthesis, and its overexpression may be neuroprotective against apoptosis [91]. One study showed that regenerating neurons of the rat facial nucleus demonstrated strong upregulation of SCD-1 whereas non-regenerating Clarke’s and Red nucleus neurons failed to, suggesting that *SCD* may play a functionally significant role in neuronal regeneration [90]. *AACS* (Acetoacetyl-CoA Synthetase) is another regulator of cholesterol and fatty acid synthesis in the CNS and is essential for normal neuronal development [92]. This gene has not been studied well with respect to spinal cord injury; however, the knockdown of *AACS* in primary neurons decreases neuronal differentiation and spine apparatus markers [92].

It is worth noting that the Long-Evans Shaker (LES) rat gives a model of chronic demyelination due to a mutation in the *MBP* gene. LES rats are congenitally dysmyelinated and yet are able to prevent axonal degeneration in vivo [93]. However, it is the exposure to inhibitory molecules and the cascade of activated phagocytes that contribute to the retraction of injured axons in SCI [94].

#### 4.4.2. Synaptic Integrity and Neurotransmission

*SYT2* (Synaptotagmin-2) is a synaptic vesicle protein that regulates fast calcium-dependent neurotransmitter release and predominates the spinal cord, unlike other members of its family [95]. The *SYT2* gene increases its expression during development and persists into adulthood, following neuronal differentiation [95]. It has not been linked to spinal cord injuries until now. *PRX* (Periaxin) is a protein-coding gene that is involved in maintaining a healthy myelin sheath; one study showed that the *PRX* gene was linked to the axon diameter of remyelination [96]. *HHIP* (Hedgehog Interacting Protein) is a vertebral cell surface protein that negatively modulates hedgehog signaling, which is required for the proper development of the mammalian neural tube from an embryo state [97]. The last gene of interest, *ZDHHC22* (Zinc Finger DHHC-Type Palmitoyltransferase 22), is responsible for regulating large conductance calcium-activated potassium channels and, interestingly, is related to tumor progression and the immune microenvironment [98,99]. *ZDHHC22* may play a neurotrophic role; however, it is yet to be studied with respect to spinal cord injury and neural regeneration.

### 4.5. Treatment of Spinal Cord Injuries and Therapeutic Considerations

Currently, the treatment for SCI involves stabilizing the spine at the time of injury, surgical decompression if needed, supportive care, management of complications, and rehabilitation. In the US, methylprednisolone is an FDA-approved drug for the treatment of SCI, although this remains controversial by international spine authorities [100]. Our results have shown that there is a complex immune response at both the acute phase 1 dpi and the chronic phase 21 dpi of SCI. Not only is there an immense innate immune response via macrophage/microglia and complement, confirmed by pathway analysis, but there is an element of auto-immune humoral immunity at play. These two systems appear to synergize with each other to propagate a destructive and chronic inflammatory state that Allison et al. described [64]. Furthermore, there are complex temporal changes to immune phenotypes and functions that warrant further investigation.

### 4.6. Limitations

Our research method focused on the gene expression at 1 and 21 dpi. Although this helped to minimize noise from transient gene expression that may not have long-term pathological significance, gene expressions are a dynamic process, and regulatory processes may occur at intermediate time points. Future research should be aimed at validating our results using functional assays and protein-level analyses to validate functional significance. Single-cell RNA sequencing should be considered to identify cell-specific gene expression changes.

## 5. Conclusions

The absent regeneration capacity of the CNS is a complex and interesting limitation of the human body that requires more research and investigation to find a solution. This study identified sustained differentially expressed genetic markers that form the foundation for future SCI research. Upregulated genes involved inflammatory and immune pathways, while downregulated genes were related to myelin sheath and neuronal synapse. The downregulated *MYOC* gene as a sole genetic marker for astrocytes may prove to hold some significance. The most novel finding of our analysis is the association of our upregulated molecular pathways with SLE and autoimmunity. Given that both SCI and SLE result in extensive immune dysfunction and a myriad of organ-system clinical manifestations, further research is warranted investigating the parallels between these two disease states. We propose that SCI is not only characterized by immune dysfunction but, over the long term, autoimmune dysregulation. This study provides a robust scaffold for functional validation and future research around immune mechanisms is warranted.

## Data Availability

The data presented in this study are available on request from the corresponding author due to privacy.

## Supplementary Materials

The following supporting information can be downloaded at: https://www.mdpi.com/article/10.3390/genes16050514/s1. Table S1: Differentially expressed genes identified comparing post-injury with control. Table S2: Top 10 enriched pathways identified in each GO annotation for the upregulated gene set. Table S3: Top 10 enriched pathways identified in each GO annotation for the downregulated gene set. Table S4: GSEA KEGG pathway analysis of up and downregulated DEGs. Table S5a: KEGG analysis of significantly upregulated genes identified in cluster analysis. Table S5b: KEGG analysis of significantly downregulated genes in cluster analysis.
