# Supplementary material for: Identification of Differentially Expressed Genes in Spinal Cord Injury
Source: Genes (Basel). 2025 Apr 28;16(5):514. doi: 10.3390/genes16050514 (PMC12111553; doi:10.3390/genes16050514)
Supplement: Supplementary file 1 [file genes-16-00514-s001.zip › Table S2.pdf]

Table S2: Top 10 enriched pathways identified in each GO annotation for the upregulated gene set

Category	Enriched Terms	Count	Fold enrichment	FDR
Biological Process	Cellular response to type II interferon	20	8.5	7.4E-10
	Cellular response to lipopolysaccharide	33	5.9	8.3E-13
	Defense response to virus	27	7.4	1.5E-12
	Innate immune response	44	6.0	1.1E-17
	Defense response to bacterium	31	6.9	8.1E-9
	Positive regulation of tumor necrosis factor production	24	10.2	9.6E-14
	Response to bacterium	31	7.6	1.3E-14
	Response to virus	16	7.4	1.6E-12
	Inflammatory response	34	5.8	7.5E-13
	Phagocytosis	15	11.4	1.1E-8
Cellular Component	CMG complex	5	26.7	1.1E-3
	Phagocytic vesicle	10	6.7	8.7E-4
	Phagocytic vesicle membrane	9	7.1	1.3E-3
	cell surface	49	3.7	2.5E-12
	External side of plasma membrane	39	4.1	5.2E-11
	Cytosol	114	1.6	2.5E-6
	Lysosome	18	3.4	1.2E-3
	Cytoplasm	156	1.5	1.5E-7
	Extracellular space	77	2.3	3.8E-10
	Extracellular region	42	2.5	7.8-6
Molecular Function	Identical protein binding	75	2.1	6.8E-7
	Protein binding	64	2.0	4.3E-5
	Integrin binding	16	5.6	4.3E-5
	Heparin binding	15	4.7	7.3E-4
	IgG binding	5	34.4	9.3E-4
	Protein homodimerization activity	32	2.3	2.6E-3
	Protein-containing complex binding	29	2.4	4.4E-3
	IgG receptor activity	4	44.1	4.8E-3
	Signalling receptor binding	20	2.8	1.0E-2
	Phosphotyrosine residue binding	7	7.6	2.0E-2
