# Supplementary material for: Identification of Differentially Expressed Genes in Spinal Cord Injury
Source: Genes (Basel). 2025 Apr 28;16(5):514. doi: 10.3390/genes16050514 (PMC12111553; doi:10.3390/genes16050514)
Supplement: Supplementary file 1 [file genes-16-00514-s001.zip › Table S3.pdf]

Table S3: Top 10 enriched pathways identified in each GO annotation for the downregulated gene set

Category	Enriched Terms	Count	Fold enrichment	FDR
Biological Process	Synaptic transmission, GABAergic II	11	44.62	2.89E-11
	Chemical synaptic transmission	16	11.26	7.03E-9
	Regulation of postsynaptic membrane potential	10	23.41	1.07E-7
	Monoatomic ion transmembrane transport	13	12.86	1.07E-7
	Regulation of presynaptic membrane potential	9	23.81	7.38E-7
	Presynaptic modulation of chemical synaptic transmission	9	18.89	4.16E-6
	Inhibitory synapse assembly	7	35.50	5.08E-6
	Neuronal action potential	8	18.72	2.89E-5
	Gamma-aminobutyric acid signalling pathway	7	25.82	3.06E-5
	Potassium ion transmembrane transport	11	8.69	6.20E-5
Cellular Component	Presynaptic membrane	28	17.09	6.43E-23
	Synapse	41	6.20	1.56E-18
	Glutamatergic synapse	41	5.56	4.84E-17
	Presynapse	27	10.09	9.85E-17
	Postsynaptic membrane	25	11.02	2.53E-16
	GABAergic synapse	21	14.52	7.58E-16
	Dendrite	32	6.37	9.22E-15
	Neuron projection	25	8.53	5.88E-14
	Plasma membrane	90	1.96	3.72E-11
	Neuronal cell body	27	5.00	6.26E-10
Molecular Function	Protein binding	47	3.21	2.36E-10
	Transmitter-gated monoatomic ion channel activity involved in regulation of postsynaptic membrane potential	10	24.18	4.14E-8
	Calcium ion binding	22	3.63	6.93E-5
	GABA-gated chloride ion channel activity	5	33.58	9.93E-4
	GABA-A receptor activity	5	31.81	9.998E-4
	Ligand gated monoatomic ion channel activity involved in regulation of presynaptic membrane potential	5	25.18	0.0022
	Inhibitory extracellular ligand gated monoatomic ion channel activity	4	40.29	0.00524
	Voltage gated potassium channel activity	6	12.09	0.0054
	Chloride channel activity	6	10.99	0.00754
	Voltage gated monoatomic ion channel activity involved in regulation of presynaptic membrane potential	4	28.44247	0.011
