# Supplementary material for: Identification of Differentially Expressed Genes in Spinal Cord Injury
Source: Genes (Basel). 2025 Apr 28;16(5):514. doi: 10.3390/genes16050514 (PMC12111553; doi:10.3390/genes16050514)
Supplement: Supplementary file 1 [file genes-16-00514-s001.zip › Table S4.pdf]

Table S4: GSEA KEGG pathway analysis for up and downregulated genes

Category	Enriched Pathways	Size	ES	NES	Nominal p-value	FDR q-value
Upregulated	KEGG Ribosome	81	0.89457	3.147857	0	0
	KEGG Systemic Lupus Erythematosus	44	0.802973	2.554543	0	0
	KEGG Cytokine Cytokine Receptor Interaction	89	0.695538	2.472493	0	0
	KEGG Antigen Processing and Presentation	37	0.806509	2.457919	0	0
	KEGG Leishmania Infection	45	0.783465	2.455322	0	0
	KEGG Complement and Coagulation Cascades	30	0.833442	2.440475	0	0
	KEGG Hematopoietic Cell Lineage	33	0.813237	2.390462	0	0
	KEGG Lysosome	106	0.643907	2.317203	0	0
	KEGG Viral Myocarditis	42	0.741087	2.299226	0	0
	KEGG Allograft Rejection	10	0.965855	2.184744	0	0
	KEGG Natural Killer Cell Mediated Cytotoxicity	65	0.647066	2.183258	0	0
	KEGG Intestinal Immune Network for IgA Production	16	0.866686	2.1529	0	0
	KEGG Autoimmune Thyroid Disease	10	0.965855	2.144486	0	0
	KEGG Primary Immunodeficiency	13	0.878809	2.122375	0	0
	KEGG Graft Versus Host Disease	9	0.965774	2.113186	0	0
	KEGG Toll Like Receptor Signalling Pathway	67	0.615779	2.098701	0	0
	KEGG Cytosolic DNA Sensing Pathway	33	0.696118	2.078349	0	5.4041127E-5
	KEGG Type I Diabetes Mellitus	16	0.794263	2.040173	0	1.0249603E-4

	KEGG Glycosaminoglycan Degradation	15	0.814951	2.020979	0	1.4837192E-4
	KEGG Asthma	7	0.983186	2.018893	0	1.4095333E-4
	KEGG B Cell Receptor Signalling Pathway	61	0.599562	1.97381	0	4.0720846E-4
	KEGG Other Glycan Degradation	14	0.792343	1.962236	0	4.32978E-4
	KEGG FC Gamma R Mediated Phagocytosis	88	0.55454	1.951994	0	4.916438E-4
	KEGG Metabolism of Xenobiotics by Cytochrome P450	22	0.710473	1.94924	0	5.110597E-4
	KEGG JAK STAT Signalling Pathway	76	0.551765	1.934295	0	7.0814515E-4
	KEGG Drug Metabolism Cytochrome P450	24	0.681898	1.883046	0.001538	0.001713
	KEGG Pathogenic Escherichia Coli Infection	44	0.578543	1.840648	0	0.003162
	KEGG RIG I Like Receptor Signalling Pathway	46	0.576684	1.830804	0.001481	0.003346
	KEGG Amino Sugar and Nucleotide Sugar Metabolism	40	0.589175	1.825968	0	0.003514
	KEGG Porphyrin and Chlorophyll Metabolism	21	0.670099	1.802985	0	0.004851
	KEGG DNA Replication	32	0.615157	1.797427	0.003021	0.005023
	KEGG Chemokine Signalling Pathway	126	0.483127	1.793424	0	0.00513
	KEGG Cell Adhesion Molecules CAMS	77	0.50724	1.767231	0.001368	0.007069
	KEGG NOD Like Receptor Signalling Pathway	39	0.556453	1.736393	0.00311	0.010369
	KEGG Pentose and Glucuronate Interconversions	10	0.767806	1.724777	0.003565	0.011597
	KEGG P53 Signalling Pathway	53	0.527789	1.718325	0.004418	0.012158
	KEGG Cell Cycle	99	0.476163	1.711575	0	0.012706

	KEGG Prion Diseases	25	0.595294	1.667004	0.009231	0.020347
	KEGG Leukocyte Transendothelial Migration	78	0.480575	1.664029	0.002778	0.020245
	KEGG Spliceosome	120	0.449883	1.64874	0	0.023658
	KEGG Starch and Sucrose Metabolism	22	0.597538	1.613748	0.031614	0.033095
	KEGG Base Excision Repair	29	0.546332	1.579915	0.009631	0.044041
	KEGG Glutathione Metabolism	40	0.508286	1.568484	0.010786	0.047429
	KEGG Folate Biosynthesis	8	0.729762	1.561131	0.042629	0.049226
	KEGG Apoptosis	66	0.464714	1.554399	0.012346	0.050846
	KEGG Chronic Myeloid Leukemia	68	0.448051	1.551521	0.009695	0.050788
Downregulated	KEGG Terpenoid Backbone Biosynthesis	13	-0.86061084	-2.232097	0	0
	KEGG Steroid Biosynthesis	14	-0.8505363	-2.2150004	0	0
	KEGG Olfactory Transduction	15	-0.7104343	-1.9704636	0	0.00443319
	KEGG Long Term Potentiation	60	-0.5144429	-1.9595041	0	0.0033248926
	KEGG Biosynthesis of Unsaturated Fatty Acids	19	-0.66509306	-1.9225096	0.0024875621	0.004802797
	KEGG Neuroactive Ligand Receptor Interaction	100	-0.4528718	-1.8833398	0	0.008287535
	KEGG Calcium Signalling Pathway	113	-0.4162672	-1.775717	0	0.029353239

113 out of 186 gene sets were upregulated in the enrichment analysis. 73 out of 186 gene sets were downregulated in the enrichment analysis.

Results are displayed up to a significant FDR q-value < 0.05
