# Supplementary material for: Identification of Differentially Expressed Genes in Spinal Cord Injury
Source: Genes (Basel). 2025 Apr 28;16(5):514. doi: 10.3390/genes16050514 (PMC12111553; doi:10.3390/genes16050514)
Supplement: Supplementary file 1 [file genes-16-00514-s001.zip › Table S5a.pdf]

Table S5a: KEGG analysis of significantly upregulated genes identified in cluster analysis

Clusters	Pathways	Count	FDR
Cluster 1	Influenza A	14	1.0E-12
	NOD-like receptor signalling pathway	10	3.6E-7
	Measles	9	1.0E-6
	Epstein-Barr virus infection	10	1.3E-6
	Hepatitis C	9	1.4E-6
	Lipid and atherosclerosis	9	9.1E-6
	Toxoplasmosis	7	2.5E-5
	Tuberculosis	8	3.1E-5
	Osteoclast differentiation	7	4.8E-5
	Toll-like receptor signalling pathway	6	2.1E-4
	Malaria	5	2.8E-4
	Coronavirus disease – COVID-19	10	4.0E-4
	Herpes simplex virus 1 infection	9	4.3E-4
	RIG-1-like receptor signalling pathway	5	4.8E-4
	Chagas disease	5	2.6E-3
	TNF signalling pathway	5	3.7E-3
	Legionellosis	4	6.6E-3
	Salmonella infection	6	9.9E-3
	Hepatitis B	5	1.1E-2
	Cystolic DNA-sensing pathway	4	1.1E-2
	NF-kappa B signalling pathway	4	1.8E-2
	Staphylococcus aureus infection	4	1.9E-2
	Kaposi sarcoma-associated herpesvirus infection	5	2.6E-2
	Human papillomavirus infection	6	2.6E-2
	African trypanosomiasis	3	2.6E-2
	Human T-cell leukemia virus 1 infection	5	3.6E-2
	Alcoholic liver disease	4	3.9E-2
	Cytokine-cytokine receptor interaction	5	4.2E-2
Cluster 2	Neutrophil extracellular trap formation	9	2.1E-7
	Leishmaniasis	7	3.1E-7
	Phagosome	8	2.7E-6
	Lipid and atherosclerosis	7	1.1e-4
	Pertussis	5	2.5E-4
	Rheumatoid arthritis	5	3.7E-4
	Tuberculosis	6	4.5E-4
	Staphylococcus aureus infection	5	4.9E-4
	Toll-like receptor signalling pathway	5	5.1E-4
	Coronavirus disease – COVID-19	8	6.9E-4
	Malaria	4	1.4E-3
	Legionellosis	4	1.9E-3
	Viral protein interaction with cytokine and cytokine receptor	4	4.1E-3
	Amoebiasis	4	5.5E-3
	Fc gamma R-mediated phagocytosis	4	5.5E-3
	Chagas disease	4	6.5E-3
	Systemic lupus erythematosus	4	7.5E-3

	Osteoclast differentiation	4	1.1E-2
	Alcoholic liver disease	4	1.2E-2
	NOD-like receptor signalling pathway	4	2.5E-2
	Complement and coagulation cascades	3	4.7E-2
Cluster 3	Ribosome	9	8.9E-10
	Coronavirus disease – COVID-19	9	2.2E-9
