# Supplementary material for: Identification of Differentially Expressed Genes in Spinal Cord Injury
Source: Genes (Basel). 2025 Apr 28;16(5):514. doi: 10.3390/genes16050514 (PMC12111553; doi:10.3390/genes16050514)
Supplement: Supplementary file 1 [file genes-16-00514-s001.zip › Table S5b.pdf]

Table S5b. KEGG analysis of significantly downregulated genes in cluster analysis

Clusters	pathways	Count	FDR
Cluster 2	Synaptic vesicle cycle	3	2.4E-3
	GABAergic synapse	2	1.8E-1
Cluster 3	Nicotine addiction	3	3.5E-4
	Neuroactive ligand-receptor interaction	4	3.5E-4
	Morphine addiction	3	7.4E-4
	GABAergic synapse	3	7.4E-4
	Retrograde endocannabinoid signalling	3	1.3E-3
